# Gender inequities in medical research funding is driving an exodus of women from Australian STEMM academia

**DOI:** 10.1111/imcb.12568

**Published:** 2022-07-05

**Authors:** Jessica G Borger, Louise E Purton

**Affiliations:** ^1^ Central Clinical School Monash University Clayton VIC Australia; ^2^ St Vincent's Institute of Medical Research Fitzroy VIC Australia; ^3^ Department of Medicine at St Vincent's Hospital The University of Melbourne Fitzroy VIC Australia

**Keywords:** academia, bias, funding, gender, STEMM, women

## Abstract

Universally, women are under‐represented in senior academic leadership in science, technology, engineering, maths and medicine (STEMM). Successful funding outcomes are a critical point in career progression, to continue both a scientist’s research but also for their retention within the STEMM workforce. A common explanation for the lower success rate of women in securing funding is that fewer women apply for funding. However, this does not adequately explain the gender inequities in funding outcomes, both in terms of fewer funded applications and also of reduced funding awarded per grant, resulting in less overall success. Gendered funding outcomes occur within academic institutions and peak funding bodies due to historical, systemic conscious and unconscious biases during peer review. As a cumulative bias over a woman’s research career, this results in women being under‐represented in STEMM and the loss of their contributions to medical research, reducing innovation through a lack of diverse workforces.

## Graphical abstract

The retention of senior women in STEMM academia is a significant issue worldwide. Our analyses of publicly available funding outcomes from the National Health and Medical Research Council of Australia (NHMRC) have revealed that gender bias is a significant contributor to the lack of retention of women.

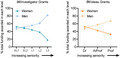

Universally, women are under‐represented in senior academic leadership in science, technology, engineering, maths and medicine (STEMM). Successful funding outcomes are a critical point in career progression, to continue both a scientist's research but also for their retention within the STEMM workforce. A common explanation for the lower success rate of women in securing funding is that fewer women apply for funding. However, this does not adequately explain the gender inequities in funding outcomes, both in terms of fewer funded applications and also reduced funding awarded per grant, resulting in less overall success. Gendered funding outcomes occur within academic institutions and peak funding bodies due to historical, systemic conscious and unconscious biases during peer review. As a cumulative bias over a woman's research career, this results in women being under‐represented in STEMM and in the loss of their contributions to medical research, reducing innovation through a lack of diverse workforces.

Academic research is historically male‐oriented. Compared with men, societally, women may be seen as lacking the brilliance necessary for discovery^1^ and are less likely to be seen as leaders in science.^2^ The oft‐touted example that even after Marie Curie was awarded her second Nobel Prize, she was refused membership of the French Académie de Sciences, exemplifies the systemic biases that have been institutionalized as policies, practices, beliefs and written or unwritten rules of behavior.^3^ Despite the increasing and almost equal recruitment of women into university degrees and early research positions, women continue to be under‐represented at senior levels of academia.^3^

Many factors contribute to the under‐representation of women in academic research. Gender stereotypes place value on male leadership traits, whilst placing expectations on women to take on the greater household or carer responsibilities, although these biases are not limited to academia and can be identified in industry, government and beyond. In academia, there are many requirements that fall more broadly on women's shoulders, including the explicit or implied obligation to sit on committees, provide pastoral care, do more service work and have more teaching commitments, which cumulatively reduce the time for their research, authoring journal articles and preparing grant applications. Further evidence demonstrates that women are under‐represented within conferences and symposiums, key events for increasing visibility and networking, and when they do present, they are less likely to be introduced with their title of doctor, one of the many biases that can go unrecognized.^4^

Peer review underlies the many processes involved in academic merit but there are concerns about its transparency and fairness. Even though women contribute more labor, they receive less credit for publications^5^ and are under‐represented as authors,^3^ with their publications being less cited than men.^6^ To compound this, on average, men cite more men, including self‐citations.^6^ Whereas journal peer review considers the research only, in which bias has not been unequivocally proven once an article is accepted for review,^7^ fellowships or investigator‐initiated grant schemes, which fund the majority of the world's medical research, also consider the quality of the applicant.

The assessment of the quality (track record) of an applicant varies according to the funding body, but usually includes how much prior funding the candidate has been awarded, their publications (quality and quantity), international reputation, invitations to speak at conferences and contributions to scientific committees, amongst other achievements. It has been recognized that there are many barriers that women face in achieving a comparable track record to men at the same academic level.^8^ While improvements in the past decade have meant that applicants are able to declare career disruptions that are to be taken into account during their track record assessments, there is evidence that these career disruptions are not being appropriately considered in Australia.^9^

There is more than enough evidence that gender gaps exist in research funding. An international meta‐analysis showed that male applicants had a 7% greater success than female applicants in grant or fellowship funding across 21 studies.^10^ As most studies of gender bias in research funding are observational, it has been unclear whether gendered outcomes derive from the peer review of the woman investigator or their proposed research. In the peer review of Early Career Researcher fellowships, the Swedish Medical Research Council assessed the applicant quality independently of the proposed research and, upon applying a gendered lens, found that women scientists with the highest productivity scored equivalently to males with the lowest productivity.^11^ Gender analysis of early career grants in the Netherlands Organization for Scientific Research also found that men applicants received more competitive “quality of researcher evaluations”, but not “quality of proposal” evaluations, resulting in men having higher success rates and there being a 4% loss of women.^12^

In 2014, the Canadian Institutes of Health Research applied a measure to combat gender disparities in peer review, dividing investigator‐initiated funding applications into two new grant programs; a fellowship program evaluating the researcher and their research as opposed to the other program which explicitly focused on the research.^13^ In the program that focused solely on the research, scores were 0.9% lower for women, remaining consistent with the traditional program, whereas when the review focus included the caliber of the investigator, the gap widened to 4%, demonstrating that gender gaps in funding are attributable to a less favorable assessment of women as investigators.^13^ This scheme was discontinued, in part due to the significant gender bias that was observed.

Australia lacks tenured positions, meaning that the majority of academic researchers in Australia rely heavily on peer‐reviewed funding to support their salary, salaries of their staff and laboratory consumables. The largest source of funding for biomedical researchers is the National Health and Medical Research Council (NHMRC), which is Australia's largest government‐supported funding body. Three years ago, the NHMRC overhauled its funding schemes and adopted a similar breakdown to Canada, with almost identical gendered results. The major people support was divided into Investigator Grants (sole Chief Investigator receives 5 years of funding for their salary plus research support packages for laboratory expenses including staff salaries), Ideas Grants (one or more Chief Investigators to fund salaries and laboratory consumables for 3–5 years) and Synergy Grants (4–10 Chief Investigators equally sharing AU$5 million over 5 years that requires mixed genders and diversity in the team composition). Ideas grants resulted in 2% lower success rates for women.^14^ These grants are considered to be scored on the quality of the research proposal and not on the researcher, but do include a score for team capability, which includes the ability of the Chief Investigator A to lead the research team in achieving the project aims. Furthermore, in the Investigator grants scheme, which assesses the quality of the researcher and which was allocated approximately 40% of the annual NHMRC funding, women had an almost 5% lower success rate.^14^ Recent data analyses by ourselves^15^ and the NHMRC^16^ demonstrate that the inequities in funding outcomes escalate as women become more senior, representing the compounding biases that women experience throughout their careers and which leverage on peer review.

There have now been three rounds of the new NHMRC grants schemes, and although the overall success rates were similar, men were disproportionately awarded a staggering 20% more grants than women, resulting in an extra AU$400 M in funding for men to continue and progress their research in only 3 years^15^ (Figure 1). Few applications were received from and awarded to non‐binary people; as a result we can only accurately compare men and women in our analyses. NHMRC do not monitor other under‐represented groups aside from Indigenous people, for whom they do not publicly report funding outcomes. As a result we cannot analyze funding outcomes for other under‐represented groups, which is important for assisting initiatives to improve diversity and inclusivity in research.

**Figure 1:**
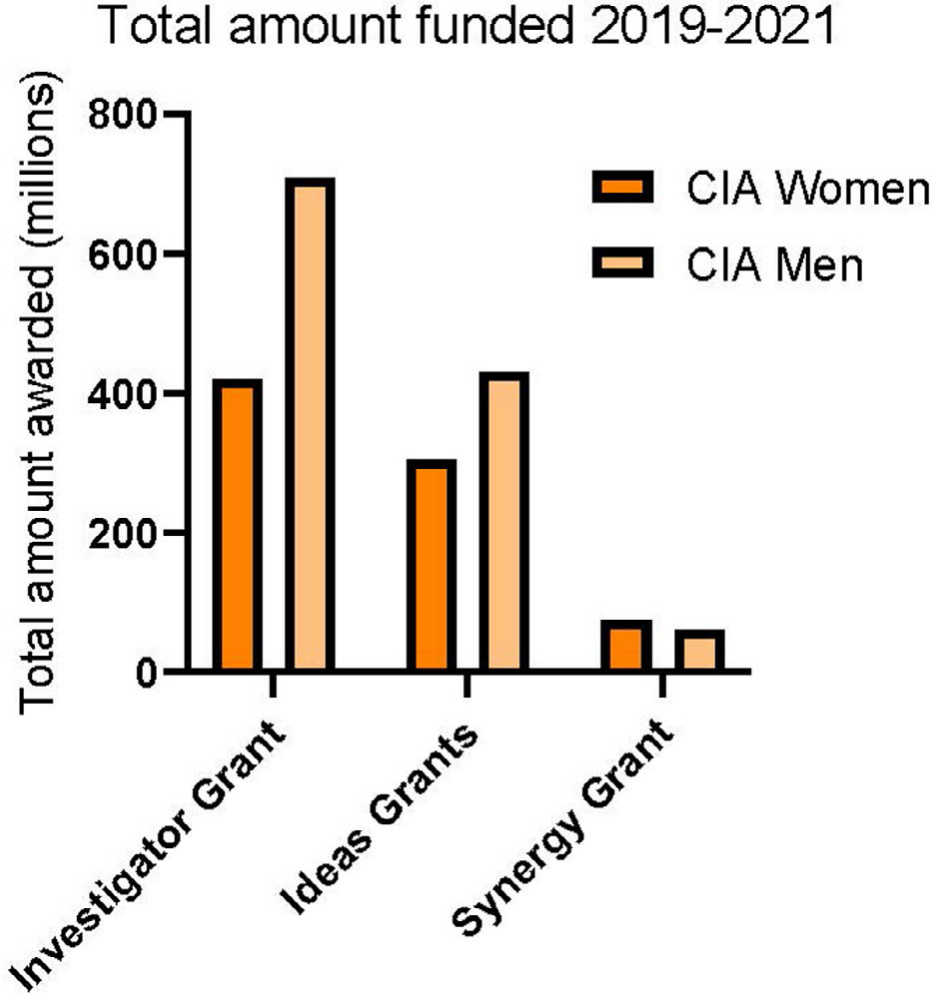
Male lead chief investigators (CIAs) were disproportionately awarded 20% more grants than women, resulting in AU$400 M more funding for men in the 3 years between 2019 and 2021. [Colour figure can be viewed at wileyonlinelibrary.com]

Although bias has been reported in European studies at the early‐career stages, the early career men and women researchers had similar funding outcomes in the first 3 years of the NHMRC Investigator grants. However, this was achieved with structural priority funding (see further below) to increase the success of women at the early career levels.^16^ It is at the early leadership stage, where researchers strive for independence and become laboratory heads, that the cumulative gendered biases start to drive inequities, to the tune of 10% in funding success.^15^ But it was not only the level of success that was reduced, the majority of women were also awarded less funding, amounting to a minimum of AU$500000 less per grant than men, substantially limiting the progression of their research compared with successful men of the same seniority.^15^ Compounding this, whereas there were similar numbers of men and women as peer reviewers of early career grants, in the more senior leadership grant levels, where there was a 20% funding deficit for women, 20% more men reviewed the grants.^16^

The NHMRC did introduce a special measure under the sexual discrimination act by providing evidence of gender disparity in their funding outcomes and introduced Structural Priority Funding (SPF) to counteract the gender disparity in 2017. SPF is an additional funding source for under‐represented minorities, primarily awarded to women, awarding funding to those who scored just below the funding cut‐off. Over 20% of successful women received this funding, with more than 30% of those at the early leadership stage, demonstrating that women scored lower than men during the peer review process, especially at the stage where they become independent laboratory heads. In the first 3 years of the Investigator grant scheme, in addition to receiving significantly fewer grants, 60% of women funded at the junior to senior laboratory head levels (L1–L3) received the lowest research support package, compared with 40% of men^16^ (Figure 2). These research support packages, which support the salaries of staff and laboratory consumables, were awarded solely based on the peer review score.

**Figure 2:**
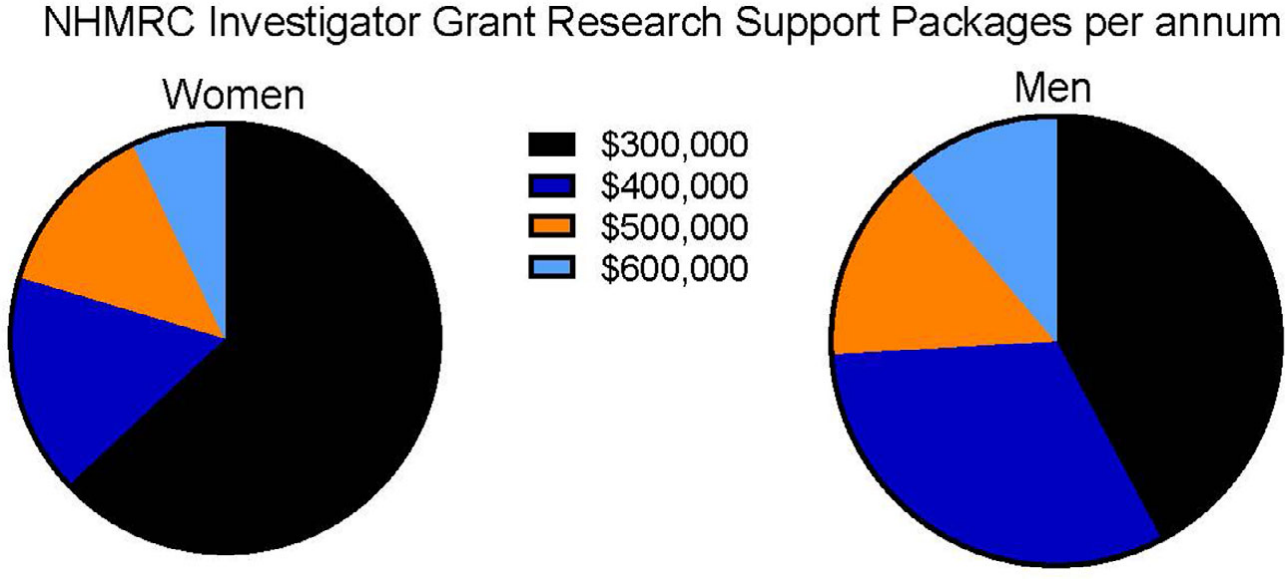
In the first 3 years of the Investigator grant scheme, 60% of women funded at the junior to senior laboratory head levels (L1–L3) received the lowest research support package. [Colour figure can be viewed at wileyonlinelibrary.com]

In 2021, of those independent women researchers who were able to retain and progress their research, at the most senior level of academic leadership (L3), only 19% of successful applicants were women.^11^ This represents a significant loss of women academic leaders and researchers to sustain Australia's innovation future.^15^ While the argument that NHMRC provided for the lack of success for women at L3 was that “more women need to apply”, it is already known that there are significantly fewer senior women in Australian STEMM academia, making it difficult to increase the numbers of women applying. Furthermore, no consideration has been given to the now recognized reduced funding that the senior women have had throughout their careers due to the effects of ongoing gender bias in peer reviewed funding and how this has impacted their productivity. Expecting women to have similar outputs to men when they have had significantly less funding and other opportunities throughout their careers is not an adequate way to account for the continuous effects of bias on careers. As a direct consequence of this, together with the restriction of each researcher to apply for a maximum of two NHMRC grant applications across the scheme per year, more senior women are likely applying for the Ideas Grants scheme, in which the track record of the applicant is not supposed to be considered during scoring. It is not possible to confirm this because the numbers of women and men in each academic level applying for Ideas Grants are not publicly available. However, in the first 3 years of the scheme, the recipients of Ideas Grants at the Professor level comprised 32% women (Figure 3b) compared with 19% of women recipients at the L3 Investigator Grant level (Figure 3a). This discrepancy is highly consistent with the 3 year averaged gendered outcomes reported in the UK when Wellcome Trust project grants were completely replaced by Investigator grants in 2010, with 26% of women receiving project grants compared with 16% of women being awarded Investigator grants.^8^

**Figure 3:**
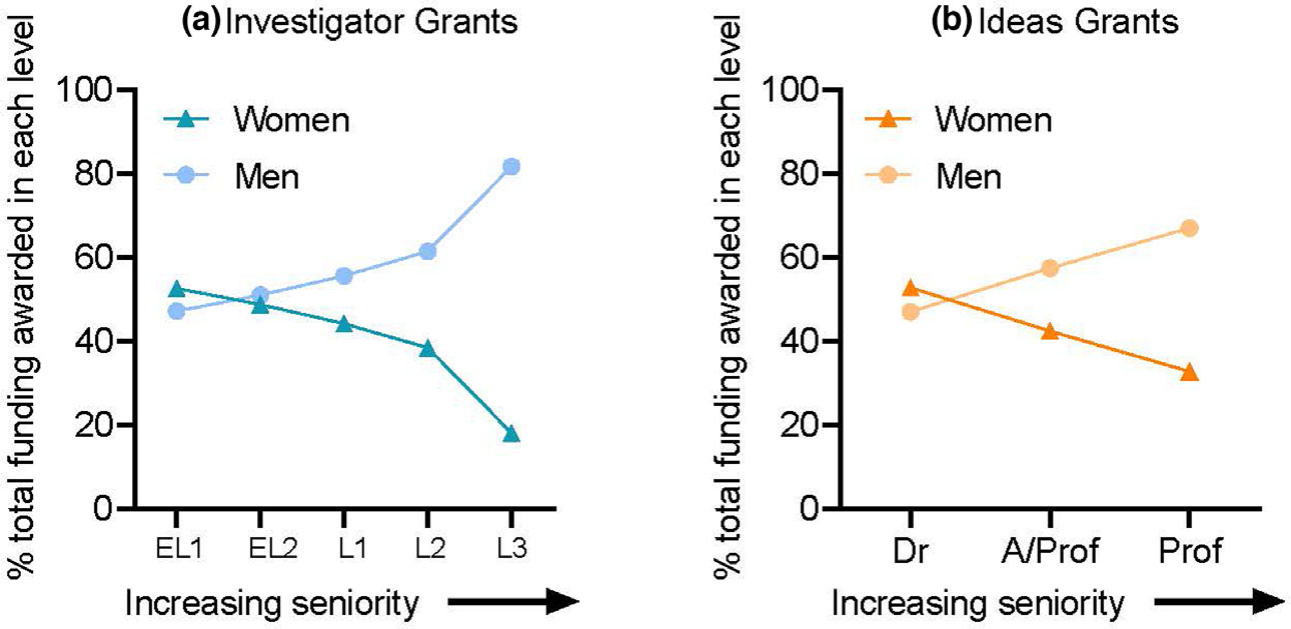
Significantly fewer women independent laboratory heads [L1–L3, Associate Professors (A/Prof) and Professors (Prof)] were successful recipients of **(a)** Investigator Grants and **(b)** Ideas Grants than men in the first 3 years of the new grant schemes. EL1 = Emerging Leadership Level 1 (<5 years post‐PhD or equivalent), EL2 = Emerging Leadership Level 2 (5–10 years post‐PhD or equivalent), L1 = Leadership Level 1 (10–15 years post‐PhD or equivalent), L2 = Leadership Level 2 (15–20 years post‐PhD or equivalent), L3 = Leadership Level 3 (> 20 years post‐PhD or equivalent). [Colour figure can be viewed at wileyonlinelibrary.com]

It is now 2022, a year that has seen the rise in advocacy for gender equity in medical research funding in social and mainstream media.^15^ Indeed, the Chief Executive Officer of the NHMRC Professor Anne Kelso, released a communique addressing the gender disparities in the NHMRC's Investigator Grant Schemes,^16^ and has delayed the commencement of the current round until January 2023 to enable the identification of appropriate interventions and policy changes to improve gendered outcomes. Although the impact of the COVID‐19 pandemic has widened the closing of the global gender gap by a generation, such that the number of years that it has been predicted are required to attain global gender equity has increased from 99.5 to 135.6 years,^17^ it has also clearly brought to the forefront the gendered burden that women experience societally and academically. We are now more empowered to leverage for change to improve funding outcomes to retain women in STEMM and work towards closing the gender gap in academia in our current generation.

## CONFLICT OF INTEREST

All authors declare that they have no conflicts of interest.

## Data Availability

The data that support the findings of this study are openly available in https://www.nhmrc.gov.au/funding/data‐research/outcomes
